# Assessment of risk factors in persistent subsolid pulmonary nodules based on CT dynamic follow-up

**DOI:** 10.1186/s12880-026-02694-5

**Published:** 2026-08-20

**Authors:** Fei Li, Jinlong Liu, Xiangyang Ren, Zihao Chen, Shenyu Yang, Yuanbo Ma, Yaman Li, Yufang Du, Xiaopeng Yang

**Affiliations:** 1 The First Affiliated Hospital of Zhengzhou University, Zhengzhou, China; 2 Zhengzhou University, Zhengzhou, China; 3 The Affiliated Hospital of Hebei University of Engineering, Hebei, China

**Keywords:** Subsolid nodules, Computed tomography, Growth prediction

## Abstract

**Background:**

This study was designed to employ CT-based dynamic follow-up and semi-automated segmentation to investigate the growth rate of SSNs and identify risk factors for their progression.

**Methods:**

We retrospectively analyzed 665 SSN patients from the NLST and 176 from the First Affiliated Hospital of Zhengzhou University. Semi-automated segmentation software measured the mean diameter, volume, and overall mean CT attenuation (m-CTA) of all nodules. Nodule mass, diameter, volume, and mass doubling time were further calculated. Based on follow-up results, nodules were divided into cancer and non-cancer groups to compare growth rates and identify the optimal method for detecting malignancy-related growth. All SSNs were further divided into growth and non-growth groups based on selected measurements for comparison of clinical and radiological features. Independent predictors of SSN growth were evaluated through Kaplan-Meier analysis and Multivariate Cox regression modeling.

**Results:**

In both datasets, significant differences (p < 0.05) were identified in all quantitative parameters including diameter, volume and mass between the cancer and non-cancer groups. Volume and its doubling time demonstrated the smallest P-values (p < 0.001), indicating that volumetric assessment provides the most sensitive measurement for detecting tumor growth. Multivariable Cox regression analysis showed that age, pleural indentation, and initial m-CTA were independent risk factors for the growth of SSNs in both datasets (all p < 0.05), while nodule type was significantly associated with growth only in the NLST dataset (p < 0.001).

**Conclusions:**

Advanced age, pleural indentation sign, and a higher initial m-CTA attenuation are independent risk factors for the growth of SSNs.

**Supplementary Information:**

The online version contains supplementary material available at 10.1186/s12880-026-02694-5.

## Introduction

Globally, lung cancer represents the most frequently diagnosed and lethal malignancy, with its incidence rapidly rising in China [1, 2]. The extensive implementation of low-dose computed tomography (LDCT) in lung disease screening has led to a marked rise in the detection of pulmonary nodules, particularly sub-solid nodules (SSNs) [3, 4]. Sub-solid nodules encompass ground glass nodules (GGNs) and part-solid nodules (PSNs), exhibiting a markedly higher malignancy rate than solid nodules [5]. Persistent SSNs, especially those demonstrating growth during follow-up, are pathologically indicative of adenocarcinoma precursors or invasive non-mucinous adenocarcinoma [6, 7].

However, SSNs often exhibit growth indolence, with varying growth rates [8, 9], making the management of such nodules still challenging. Current pulmonary nodule management guidelines indicate that most SSNs require subsequent follow-up [10, 11]. Although radiological monitoring of SSNs during follow-up examinations is relatively straightforward to implement, changes in these nodules occur over long time intervals. Consequently, it is generally difficult to identify nodules with interval growth and potential malignant transformation during long-term follow-up [12]. Therefore, a key challenge in clinical practice is to accurately distinguish high-risk SSNs with growth and malignant potential from a large number of indolent SSNs [13, 14]. Addressing this requires a deeper understanding of SSN growth patterns and the identification of reliable predictive factors for progression.

Based on this, the study aims to systematically investigate the natural course, growth rate, and independent risk factors for the growth of persistent SSNs during long-term follow-up by integrating CT imaging features with a semi-automated segmentation method. This research seeks to provide evidence for individualized follow-up and management of SSNs.

## Methods

### Patient selection

This research utilized a retrospective design, incorporating data from two sources: the National Lung Screening Trial (NLST) for SSNs identified from August 2002 to December 2009, and a local institutional cohort comprising SSN cases collected between July 2019 and May 2025.

Inclusion criteria were as follows: (1) a minimum of two thin-slice CT scans (≤ 2.5 mm) obtained at least 6 months apart; (2) at least one sub-solid nodule; and (3) SSNs with a diameter of 5–30 mm on the initial CT image. Exclusion criteria were as follows: (1) only a single CT examination; (2) follow-up period < 6 months from initial CT scan; (3) CT images showing significant respiratory or metallic artifacts; and (4) patients undergoing neoadjuvant therapy or radiotherapy or chemotherapy. If a patient had multiple SSNs, the “largest nodule priority” principle was applied, and only the largest SSN meeting the criteria was selected for analysis.

In total, 665 patients with SSNs from the NLST dataset and 176 patients with SSNs from our institutional cohort were included in the final analysis. As this was a retrospective study based on existing datasets, no prospective sample size calculation was performed. The final sample size was determined by the availability of eligible cases and was considered adequate for the planned statistical analyses.

### CT examination

This retrospective study included CT examinations acquired using different scanners and acquisition protocols. To minimize the influence of technical variability on quantitative assessment, only thin-section CT examinations with a slice thickness ≤ 2.5 mm reconstructed using standard algorithms were included. All CT images were reviewed and processed using the same semi-automated segmentation software (uAI-ChestCare, Shanghai United Imaging Intelligence, China). The scanning parameters were as follows: collimation 0.625–1.25 mm, tube current 120–200 mA, tube voltage 120 kVp, FOV 350–400 mm, and matrix 512 × 512 or 1024 × 1024. Reconstruction slice thickness and interval ranged from 1 to 2.5 mm.

### Clinical and radiological analysis

Patient clinical data were retrieved from the institutional picture archiving and communication system (PACS), including age, gender, smoking history, follow-up period, and follow-up confirmation results. Imaging features comprised initial nodule type, nodule location, spiculation, lobulation, air bronchogram, pleural indentation, and vacuole sign.

All imaging features were independently assessed by two chest radiologists (one junior radiologist with 3 years of diagnostic experience and one senior radiologist with 15 years of diagnostic experience) based on the initial thin-slice CT images. Inter-observer agreement for categorical imaging features was evaluated using Cohen’s kappa coefficient (κ). Disagreements were resolved through consultation, and GGNs and PSNs were identified on the initial thin-slice CT.

### Nodule segmentation

The same semi-automated segmentation software (uAI-ChestCare) was utilized to perform pulmonary nodule detection and segmentation on both baseline and follow-up thin-slice CT images. When the ROI included vessels or air cavities, the nodule margins were manually corrected by radiologists. After segmentation, the software automatically calculated and recorded the mean diameter, volume, and mean CT attenuation (m-CTA) of each nodule. The mass of each nodule (M, mg) was computed as follows [15]: M = V × (A + 1000) / 1000, where V represents the nodule volume (mm³) and A represents the mean CT attenuation value (HU).

To evaluate segmentation reproducibility, 30 randomly selected SSNs were independently segmented by two experienced radiologists, and the intraclass correlation coefficient (ICC) was calculated to assess inter-observer agreement of quantitative measurements. The ICC values ranged from 0.85 to 0.96, indicating good to excellent reproducibility of the segmentation results.

### Nodule growth analysis

Based on final clinical diagnosis, SSNs in the NLST and our institutional dataset were classified into cancer and non-cancer groups. Cancer status was determined based on pathological confirmation when available, whereas cases without pathological confirmation were classified according to longitudinal imaging follow-up and clinical evaluation. For each SSN, diameter, volume, and mass growth rates were individually computed based on longitudinal measurements. Subsequently, the volume doubling time (VDT), mass doubling time (MDT), and diameter doubling time (DDT) for each nodule were calculated using the modified Schwartz formula [16]. The doubling time (DT) was defined as DT = (log₂ × T) / log (X_f_ / X_i_), where X_f_ and X_i_ represent the final and initial measurements (volume, mass, or diameter), respectively, and T is the time interval (in days) between consecutive CT examinations. Based on this, the three relative ratios were compared between the cancer and non-cancer groups to determine the optimal method for detecting nodule growth. Finally, all SSNs were categorized into growth and non-growth groups based on the selected quantitative metrics.

### Statistical analysis

Continuous variables following a normal distribution were reported as mean ± standard deviation (SD), whereas non-normally distributed variables were expressed as median (range). Categorical variables were reported as frequency (percentage). For comparing continuous variables between the growth and non-growth groups, the t-test and Mann-Whitney U test were utilized. Differences in categorical variables were evaluated using the χ² test or Fisher’s exact test, depending on applicability. The optimal cutoff points for quantitative SSNs characteristics were established through analysis with X-tile v.3.6.1. The Kaplan-Meier analyses with log-rank tests were used to compare differences in growth time of SSNs among subgroups. Variables showing significant associations with SSN growth in univariate analyses were subsequently entered into multivariate Cox proportional hazards regression models to identify independent predictors of SSN growth.

All statistical analyses were performed using SPSS v.27.0 (IBM Corporation). Differences were considered statistically significant at *p* < 0.05.

## Results

### Growth-to-variability ratio

The NLST dataset consisted of 665 patients (665 SSNs), including 548 GGNs and 117 PSNs. Our institutional cohort consisted of 176 patients (176 SSNs), including 118 GGNs and 58 PSNs. To compare the performance of different quantitative approaches for detecting SSN progression, all nodules were stratified into cancer and non-cancer groups according to the final clinical diagnosis. Growth rates and doubling times for nodule diameter, volume, and mass were individually computed and are presented in Table 1. In the NLST dataset, all measured values differed significantly between the cancer and non-cancer groups (all *P* < 0.001). In the institutional dataset, significant differences in all measurements between cancer and non-cancer groups were also observed (*P* < 0.05), with volume showing the most significant difference (*P* < 0.001). Furthermore, the volume doubling time was shortest among malignant nodules, suggesting that volumetric analysis offers superior sensitivity in identifying growth.

**Table 1:** Comparative analysis of growth rates in the NLST and institutional cohorts

Type	NLST			Institutional		
	Cancer (*n* = 107)	Non-cancer (*n* = 558)	*p*-value	Cancer (*n* = 106)	Non-cancer (*n* = 70)	*p*-value
Diameter						
Rate	0.36 ± 0.35	0.18 ± 0.17	< 0.001	0.21 ± 0.19	0.13 ± 0.08	0.004
DDT	4360.59 (407.98, 29689.48)	5740.33 (217.26, 28233.63)	< 0.001	4370.05 (2547.15, 25283.76)	4941.40 (698.19, 29550.89)	< 0.001
Volume						
Rate	1.42 ± 1.94	0.23 ± 0.20	< 0.001	0.57 ± 0.96	0.24 ± 0.15	< 0.001
VDT	1567.77 (107.19, 9561.92)	3178.40 (421.35, 14659.11)	< 0.001	1762.53 (191.28, 7795.21)	2520.93 (389.29, 7909.73)	< 0.001
Mass						
Rate	1.05 ± 1.33	0.24 ± 0.20	< 0.001	0.35 ± 0.21	0.24 ± 0.17	0.020
MDT	2894.10 (174.69, 18067.42)	5328.26 (708.63, 28719.67)	< 0.001	1682.61 (267.36, 9534.02)	2510.20 (235.91, 9808.11)	0.002

**Fig. 1:**
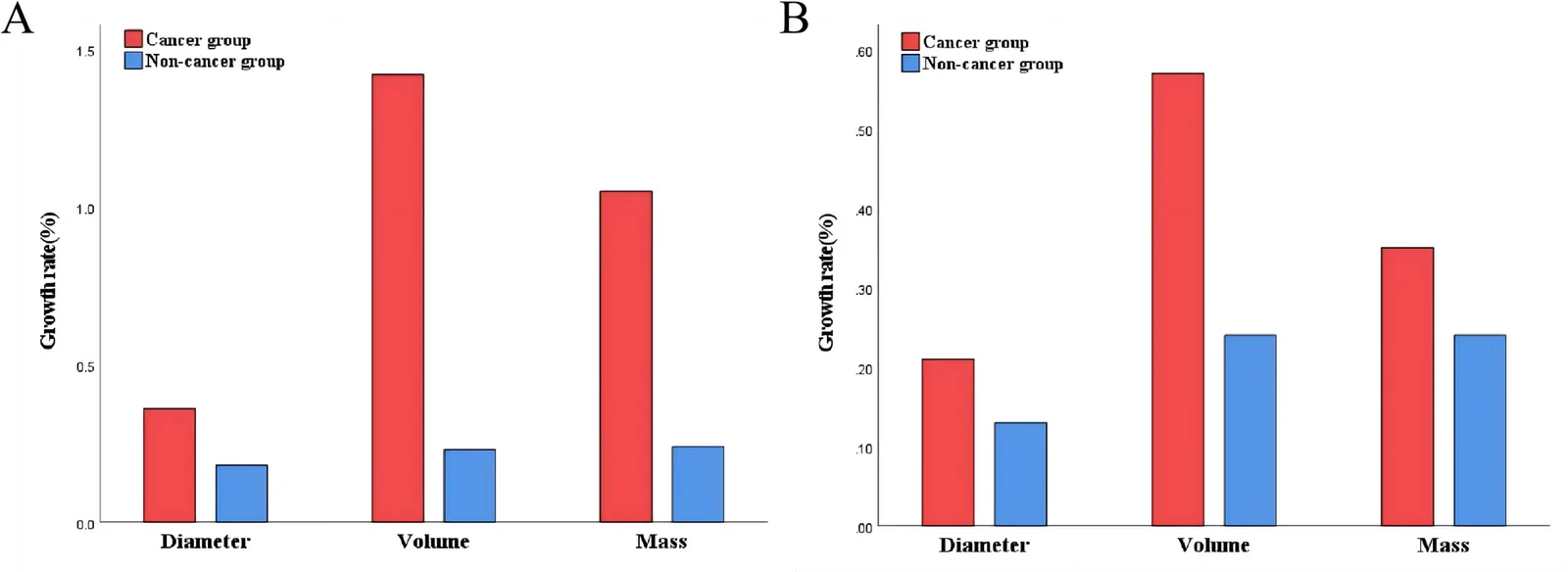
Progression of diameter, volume, and mass in SSNs. (**A**) NLST dataset. (**B**) Institutional dataset

The average inter-scan interval for evaluated SSNs was 746 days (range: 342–1026) in the NLST cohort and 505 days (range: 185–1665) in the institutional cohort. Throughout the observation period, malignant nodules in the NLST cohort exhibited mean diameter, volume, and mass increases of 26%, 142%, and 105%, respectively. In contrast, non-malignant nodules showed substantially lower growth, with average increments of 18%, 23%, and 24% for the same parameters. In our institutional dataset, the mean increases in diameter, volume, and mass for cancer SSNs were 21%, 57%, and 35%, respectively, while for non-cancer SSNs, the mean increases were 13%, 24%, and 23%. Volumetric growth rate demonstrated greater sensitivity than diameter or mass changes in differentiating malignant from benign nodules (Fig. 1). A volume increase exceeding 25% was associated with a significant growth trend in SSNs and a higher likelihood of progression to lung cancer.

### Clinical and radiologic characteristics of growth and non-growth nodules

Based on these findings, SSN growth was defined as a volumetric increase greater than 25% from baseline to the final CT examination.

The NLST dataset included 665 patients, comprising 388 in the non-growth group and 277 in the growth group (Table 2). Clinical feature analysis showed that patients exhibiting nodule growth were older than those in the non-growth group (*P* = 0.041), and those with a history of smoking were more likely to experience nodule growth (*P* = 0.014). No significant differences were identified between the two groups regarding gender distribution (*P* = 0.537) or follow-up duration (*P* = 0.894). Concerning imaging characteristics, nodules in the growth group exhibited significantly larger initial diameter, volume, mass, and higher m-CTA values compared to the non-growth group (*P* < 0.05). PSNs were significantly more prevalent in the growth group (26.4%) than in the non-growth group (11.3%) (*P* < 0.001). The tendency for growth increased when nodules exhibited irregular morphology (*P* = 0.005). Other imaging features, such as the air bronchogram sign and pleural indentation sign, were also associated with nodule growth (*P* < 0.05).

This institutional dataset included 176 patients, comprising 94 in the non-growth group and 82 in the growth group. Clinically, patients in the growth group were significantly older and exhibited a higher rate of smoking history relative to the non-growth group (*P* < 0.001). In contrast, no significant differences were observed in follow-up duration or gender distribution (*P* = 0.337 and *P* = 0.777, respectively). Imaging characteristics revealed that the growth group exhibited significantly larger initial nodule diameter, initial volume, m-CTA values, and PSN proportion compared to the non-growth group (*P* < 0.05). Additionally, radiographic features including lobulation, spiculation, pleural indentation sign, and air bronchogram sign showed significant correlations with nodule growth (*P* < 0.05). Inter-observer agreement analysis demonstrated good agreement between the two radiologists for categorical imaging features.

**Table 2:** Clinical and nodule characteristics in the NLST and Institutional datasets

Characteristics	NLST			Institutional		
	Growth group (*n* = 277)	Non-growth group (*n* = 388)	*P* value	Growth group (*n* = 82)	Non-growth group (*n* = 94)	*P* value
**Clinical characteristics**						
Age	62.7 (55, 74)	62.0 (55, 74)	0.041	57.9 (19, 73)	52.0 (25, 81)	< 0.001
Sex			0.537			0.777
Female	159 (57.4%)	232 (59.8%)		48 (58.5%)	57 (60.6%)	
male	118 (42.6%)	156 (40.2%)		34 (41.5%)	37 (39.4%)	
Smoking history			0.014			< 0.001
Yes	201 (72.6%)	313 (80.7%)		40 (48.8%)	72 (76.6%)	
No	76 (27.4%)	75 (19.3%)		42 (51.2%)	22 (23.4%)	
Follow-up time	744.3 (342.0, 1026.0)	746.6 (350.0, 1014.0)	0.894	525.8 (189.0, 1665.0)	486.6 (185.0, 1490.0)	0.337
**Nodular characteristics**						
Type			< 0.001			0.001
GGN	204 (73.6%)	344 (88.7%)		45 (54.9%)	73 (77.7%)	
PSN	73 (26.4%)	44 (11.3%)		37 (45.1%)	21 (22.3%)	
Initial diameter (mm)	9.1 (5.1, 27.6)	7.1 (5.0, 24.3)	< 0.001	9.1 (5.7, 21.1)	8.1 (5.5, 14.7)	0.005
Initial volume (mm^3^)	529.0 (22.5, 9432.5)	203.9 (28.5, 3930.0)	0.006	426.5 (99.7, 2793.4)	347.3 (94.4, 1475.3)	0.028
Initial mass (mg)	321.5 (13.0, 5469.5)	109.4 (13.7, 3018.1)	0.005	160.9 (9.6, 1396.6)	154.8 (26.7, 1150.8)	0.942
Initial m-CTA (HU)	-397.9 (-843.2, -229.3)	-423.5 (-890.1, -271.2)	0.017	-598.8 (-809.4, -260.2)	-635.5 (-812.3, -342.0)	0.036
VDT (days)	710.0 (107.2, 2299.6)			872.2 (191.3, 4013.6)		
MDT (days)	1658.2 (174.7, 15439.4)			1369.9 (236.0, 9808.1)		
Irregular			0.005			0.053
No	218(78.7%)	337 (86.9%)		58 (70.7%)	78 (83.0%)	
Yes	59 (21.3%)	51 (13.1%)		24 (29.3%)	16 (17.0%)	
Lesion location			0.163			0.069
Right upper lobe	62 (22.4%)	109 (28.1%)		23 (28.0%)	25 (26.6%)	
Right middle lobe	39 (14.1%)	36 (9.3%)		11 (13.4%)	7 (7.4%)	
Right lower lobe	58 (20.9%)	83 (21.4%)		11 (13.4%)	25 (26.6%)	
Left upper lobe	56 (20.2%)	86 (22.2%)		23 (28.0%)	22 (23.4%)	
Left lower lobe	62 (22.4%)	74 (19.1%)		14 (17.1%)	15 (16.0%)	
Morphologic features						
Lobulated sign	30 (10.9%)	38 (9.8%)	0.652	37 (45.1%)	19 (20.2%)	< 0.001
Spiculated sign	35 (12.6%)	33 (8.5%)	0.083	21 (25.6%)	8 (8.5%)	0.002
Pleural indentation	68 (24.5%)	61 (15.7%)	0.005	33 (40.2%)	22 (23.4%)	0.016
Air bronchogram	33 (11.9%)	23 (5.9%)	0.006	17 (20.7%)	6 (6.4%)	0.005
Vacuole sign	19 (6.9%)	26 (6.7%)	0.936	11 (13.4%)	7 (7.4%)	0.192

Additional subgroup analyses were performed according to nodule subtype (GGNs and PSNs). In the NLST cohort, the GGN subgroup showed significant differences between the growth and non-growth groups in age, initial diameter, volume, mass, m-CTA, irregular margin, pleural indentation sign, and air bronchogram sign (all *P* < 0.05). In the PSN subgroup, only initial m-CTA showed a significant difference between the growth and non-growth groups (*P* < 0.001). In the institutional cohort, significant differences between the growth and non-growth groups in the GGN subgroup were observed for age, smoking history, lobulation, spiculation, and pleural indentation sign (all *P* < 0.05), whereas no significant differences were observed in the PSN subgroup. Detailed results are presented in Supplementary Tables S1 and S2.

### Risk factors for SSN growth

**Fig. 2:**
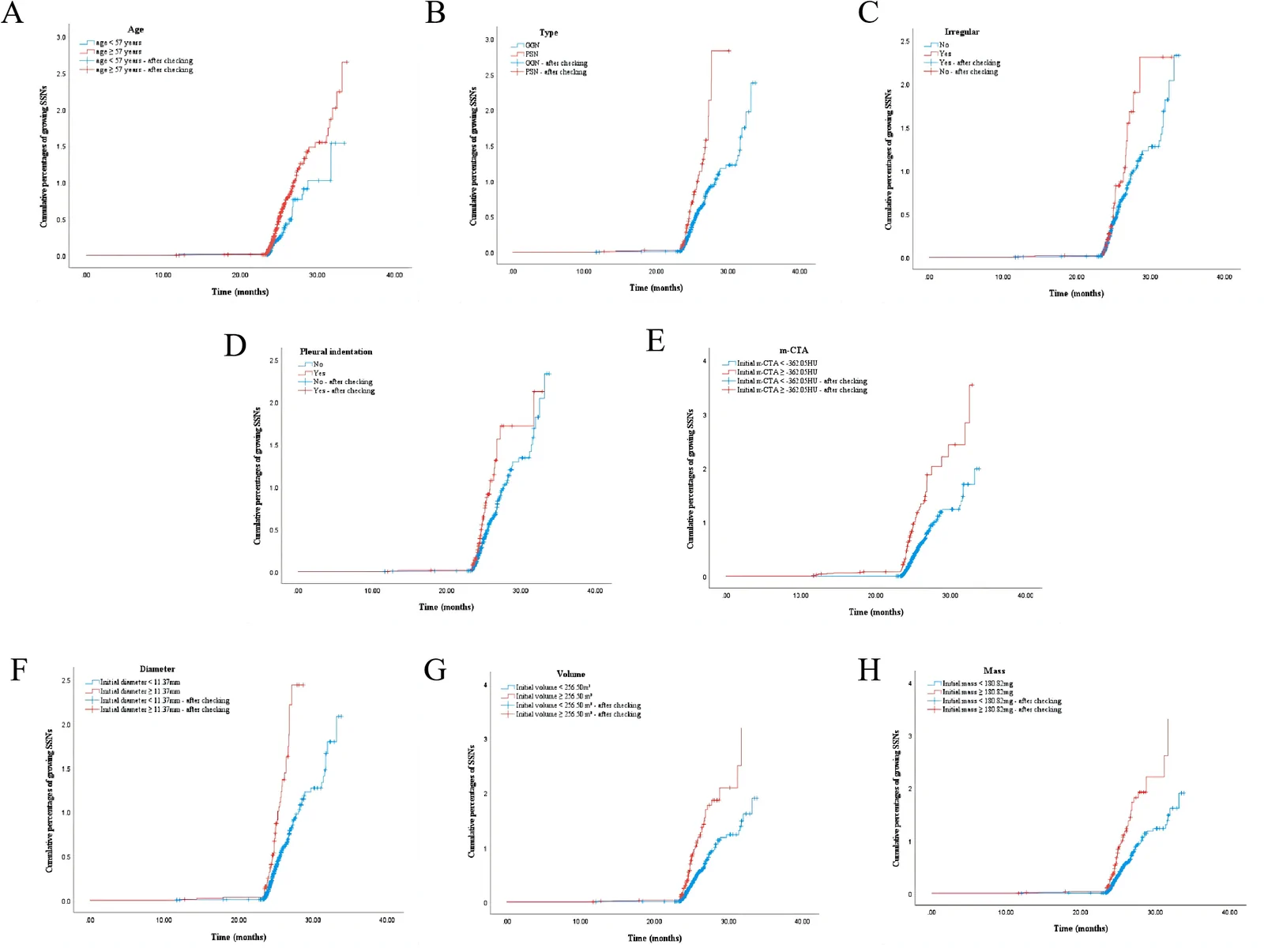
Kaplan-Meier analysis of SSN growth by predictors: (**A**) Age. (**B**) Nodule type. (**C**) Morphology. (**D**) Initial m-CTA. (**E**) Pleural indentation. (**F**) Initial mass. (**G**) Initial diameter. (**H**) Initial volume

**Fig. 3:**
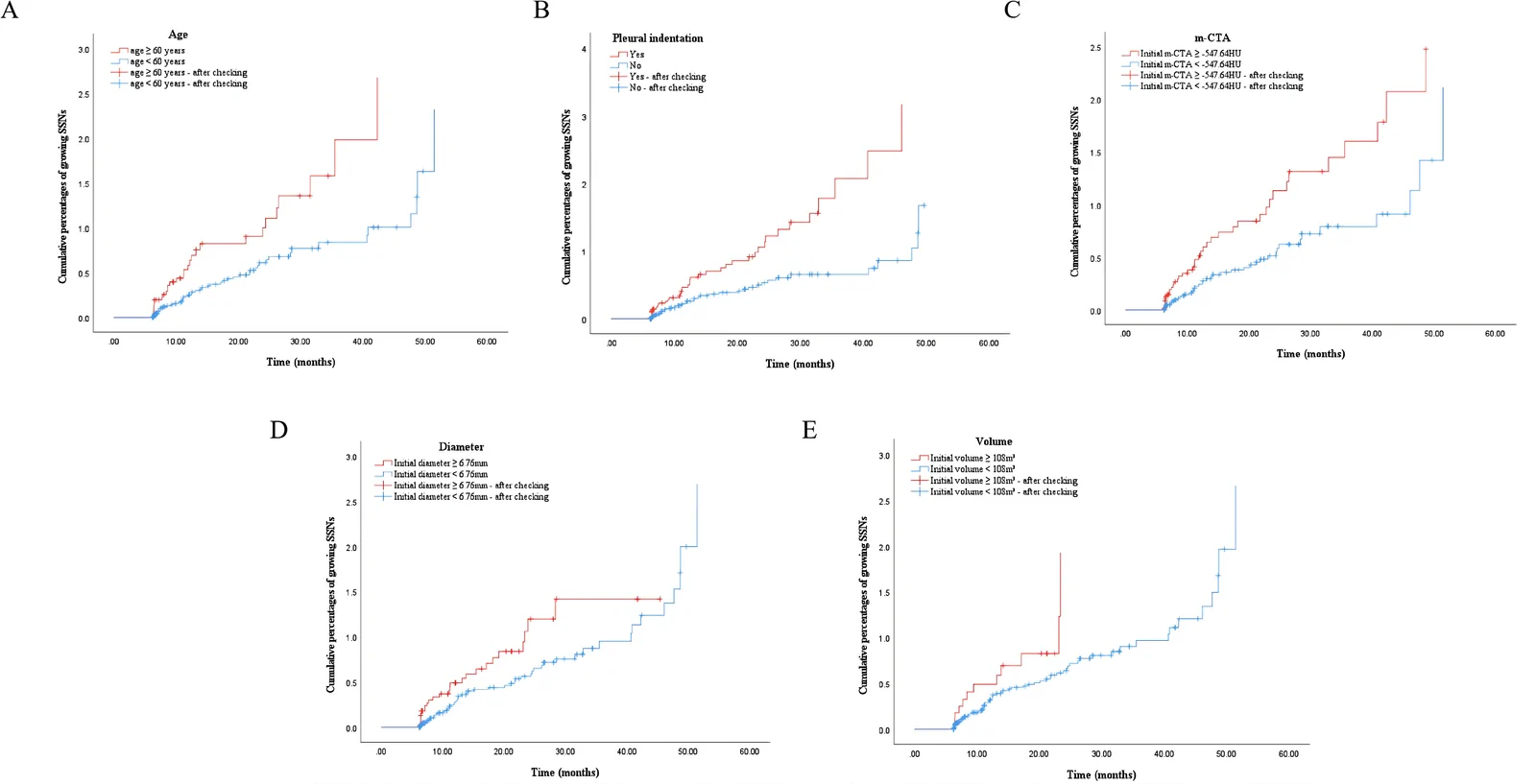
Kaplan-Meier analysis of SSN growth by predictors: (**A**) Age. (**B**) Initial m-CTA. (**C**) Pleural indentation. (**D**) Initial diameter. (**E**) Initial volume

Kaplan-Meier survival analysis, supplemented by log-rank testing, indicated that in the NLST dataset, nodule morphology (*P* = 0.018), smoking history (*P* = 0.003), nodule type (*P* < 0.001), pleural indentation sign (*P* < 0.001), age ≥ 57 years (*P* = 0.002), initial mean diameter ≥ 11.37 mm (*P* < 0.001), initial volume ≥ 256.5 mm³ (*P* < 0.001), initial m-CTA ≥-362.5 HU (*P* < 0.001), and initial mass ≥ 180.82 mg (*P* < 0.001) were significantly associated with increased cumulative growth rates (Fig. 2). In our institutional dataset validation, smoking history ((*P* = 0.015), pleural indentation sign (*P* < 0.001), age ≥ 60 years (*P* < 0.001), initial mean diameter ≥ 6.76 mm (*P* = 0.022), initial volume ≥ 108 mm³ (*P* = 0.018), and initial m-CTA ≥-547.64 HU (*P* < 0.001) also demonstrated significantly increased cumulative growth rates (Fig. 3).

After adjustment for potential confounding factors, multivariate Cox proportional hazards regression identified age ≥ 57 years (HR = 1.566, *P* = 0.005), nodule type (HR = 1.737, *P* = 0.002), pleural indentation sign (HR = 1.895, *P* < 0.001), and baseline m-CTA ≥ -362.5 HU (HR = 2.027, *P* < 0.001) as independent predictors of SSN growth in the NLST cohort (Table 3). In the institutional cohort, age ≥ 60 years (HR = 2.104, *P* = 0.002), pleural indentation sign (HR = 2.145, *P* < 0.001), and initial m-CTA ≥ -547.64 HU (HR = 1.719, *P* = 0.019) were also identified as independent predictors of SSN growth after adjustment for potential confounding factors (Table 4).

**Table 3:** Results of univariate and multivariate analyses predicting SSN growth in the NLST dataset

Characteristics	Univariate analysis	Multivariate analysis		
	*P* value	HR	95%-CI	*P* value
Sex	0.424			
Age	0.002	1.566	1.143–2.146	0.005
Smoking history	0.003			
Type	< 0.001	1.737	1.140–1.897	0.002
Lesion location	0.899			
Shape	0.018			
Lobulated	0.231			
Spiculation	0.135			
Pleural indentation	< 0.001	1.895	1.460–2.647	< 0.001
Air bronchogram	0.243			
Vacuole	0.730			
Initial diameter ≥ 11.37 mm	< 0.001			
Initial volume > 256.5mm^3^	< 0.001			
Initial m-CTA ≥ -362.5HU	< 0.001	2.027	1.539–2.671	< 0.001
Initial mass ≥ 180.82 mg	< 0.001			

**Table 4:** Results of univariate and multivariate analyses predicting SSN growth in the institutional dataset

Characteristics	Univariate analysis	Multivariate analysis			
	*P* value	HR		95%-CI	*P* value
Sex	0.694				
Age	< 0.001	2.104	1.306–3.387		0.002
Smoking history	0.015				
Type	0.250				
Lesion location	0.577				
Shape	0.936				
Lobulated	0.414				
Spiculation	0.113				
Pleural indentation	< 0.001	2.145	1.374–3.348		< 0.001
Air bronchogram	0.800				
Vacuole	0.400				
Initial diameter ≥ 6.76 mm	0.022				
Initial volume > 108.00mm^3^	0.018				
Initial m-CTA ≥ -547.64HU	0.001	1.719	1.092–2.704		0.019
Initial mass ≥ 58.93 mg	0.133				

## Discussion

Lung cancer ranks among the malignant tumors with the highest global incidence [17,18], and its early detection and diagnosis are crucial for improving patient prognosis [19]. Persistent SSNs, as common early manifestations of lung cancer, typically grow slowly, making it challenging to accurately identify nodules with potential malignant risk during long-term follow-up [20]. Consequently, accurately distinguishing stable SSNs from those with any detectable growth is crucial for guiding subsequent clinical management. Based on the NLST and our institutional dataset, this study conducted a systematic comparison of diameter, volume, and mass measurements for assessing SSN growth, demonstrating the superior sensitivity of volumetric analysis. On this basis, SSNs in both datasets were classified into progression and stability groups based on volume change to further explore the risk factors associated with SSN growth. Multivariate analysis showed that advanced age, higher initial m-CTA values, and pleural indentation sign were established as independent predictors of growth in SSNs. These findings suggest that quantitative imaging parameters and morphological characteristics provide complementary information for SSN growth assessment. Compared with previous studies focusing mainly on individual clinical or morphological predictors, this study integrates longitudinal quantitative CT parameters with clinical and imaging characteristics and further validates the identified predictors in an external cohort. The combination of m-CTA and pleural indentation may improve risk stratification of persistent SSNs.

In this study, 41.6% and 53.4% of SSNs in the two datasets showed growth during the follow-up period, respectively. This observed proportion exceeds rates documented in prior research [21,22]. This may be related to the study’s criteria for defining SSN growth as a volume change exceeding 25%. Compared with the criteria of a diameter increase of ≥ 2 mm used in most previous studies [23,24], the volume metric reflects longitudinal changes of nodules across three dimensions, thus allowing more sensitive detection of subtle longitudinal changes in SSNs. Furthermore, volumetric growth rates observed during follow-up were significantly higher than those of diameter and mass, further indicating that volume had higher sensitivity and accuracy in reflecting the growth of SSNs. This criterion is also consistent with recommendations from the British Thoracic Society guidelines [25]. Additionally, the use of semi-automated segmentation for diameter and volume measurements in this study effectively reduced labor and time expenditures. However, nodule growth should not be considered a standalone surrogate marker of malignancy. Some benign SSNs may also demonstrate progressive enlargement during follow-up. Therefore, volumetric growth assessment should be interpreted in combination with clinical risk factors and morphological characteristics rather than being considered an independent indicator of malignancy.

This study found that the m-CTA value associated with nodular density is an independent risk factor for subsequent SSN growth. This result is consistent with studies by Gao et al. and He et al. [26,27], which also reported that higher m-CTA values were significantly associated with more rapid progression and malignancy in SSNs. The reason may be that the m-CTA value reflects the average density of nodules, and a higher density often indicates an elevated tumor cell count, a dense arrangement, or more abundant blood supply and metabolic activity. These biological characteristics together prompt SSNs with a higher m-CTA value to exhibit a stronger growth tendency [28,29].

Multivariate Cox regression confirmed that advanced age and pleural indentation sign were significant factors for SSN growth in both datasets, consistent with findings from multiple prior studies [22,24, 30,31]. Advanced age was significantly associated with an increased risk of SSN growth. A meta-analysis involving 19 original studies also showed that patients over 65 years old had a 2.26 times greater risk of nodule growth compared to younger patients [32]. The research by Yuan et al. [33] further found that the pleural indentation sign served as an independent predictor of growth in SSNs. This sign often indicates the presence of fibrous adhesions between the nodule and the pleura, which may reflect early pleural involvement to a certain extent, suggesting an association with the growth and invasive behavior of the nodule [34]. Additionally, in the NLST dataset, nodule type was also independently associated with SSN growth, although this association was not reproduced in our institutional dataset. This discrepancy may be related to differences in sample size, patient characteristics, and nodule composition between the two cohorts. Furthermore, subgroup analyses according to nodule subtype demonstrated that growth-associated characteristics differed between GGNs and PSNs. GGNs showed significant differences in multiple clinical and radiologic characteristics between growth and non-growth groups, whereas fewer significant differences were observed in PSNs. The limited number of PSNs, particularly in the institutional cohort, may have reduced the statistical power to detect potential associations within this subgroup. These findings suggest that different SSN subtypes may exhibit distinct growth patterns, which may partly explain the heterogeneous association between nodule type and SSN growth across cohorts. Future studies with larger and more balanced cohorts are needed to further clarify the influence of nodule subtype on SSN growth. From a clinical perspective, these findings may help identify patients with persistent SSNs who require closer surveillance during follow-up.

This study has several limitations. First, this study was retrospective in design and involved two independent cohorts, resulting in unavoidable variations in CT scanners, acquisition protocols, and reconstruction thickness, which may have influenced the quantitative assessment of SSNs. Although standardized reconstruction algorithms and a unified semi-automated segmentation platform were applied, residual technical heterogeneity may still exist. Future prospective multicenter studies with standardized CT acquisition protocols are warranted to further improve the reliability of quantitative SSN assessment. Second, subgroup analysis could not be performed based on pathological confirmation, and the volume growth threshold used to define SSN growth was not directly validated against pathological outcomes. Further studies incorporating pathologically confirmed cohorts are needed to validate imaging-based growth criteria. Third, morphological features in this study were mainly assessed using conventional binary classifications, without detailed stratification of morphological patterns. In addition, a comprehensive prediction model integrating clinical factors, morphological features, and quantitative parameters was not developed. Future studies incorporating more detailed imaging feature analysis and integrated predictive models are warranted to further improve SSN risk stratification. Fourth, although subgroup analyses according to nodule subtype were performed, the imbalanced distribution of PSNs and GGNs, particularly the limited number of PSNs, may have reduced the statistical power for detecting subtype-specific differences. Future studies with larger and more balanced cohorts are warranted to further validate the growth characteristics of different SSN subtypes. Finally, the semi-automated measurement software used in this study depended on specific segmentation algorithms, potentially affecting measurement consistency and reproducibility.

## Conclusion

Compared to assessments based on diameter or mass, the volumetric growth rate proved to be a more sensitive indicator for evaluating the progression of lung cancer-associated SSNs. Based on the criterion of volume growth, this study further explored the independent risk factors influencing the growth of persistent SSNs. Multivariate analysis showed that advanced age, higher m-CTA values, and the presence of pleural indentation were significant predictors of subsequent SSN growth. The results indicated that SSNs in elderly patients, those with a higher m-CTA value, and those with pleural indentation were more likely to grow. Therefore, close monitoring of the aforementioned imaging and clinical features is essential in clinical management, and patients should be stratified according to these risk factors to facilitate appropriate surveillance and management. The integration of semi-automated segmentation with longitudinal CT follow-up provides an efficient and reproducible approach for quantitative SSN monitoring, supporting individualized surveillance strategies and risk stratification in clinical practice.

## Supplementary Information

Below is the link to the electronic supplementary material.

Supplementary Material 1

## Data Availability

The datasets used and/or analysed during the current study are available from the corresponding author on reasonable request.

## Abbreviations

SSNs: Sub-solid nodules
GGNs: Ground glass nodules
PSNs: Part-solid nodules
NLST: The National Lung Screening Trial
m-CTA: Overall mean CT attenuation
ICC: Intraclass correlation coefficient
VDT: Volume doubling time
MDT: Mass doubling time
DDT: Diameter doubling time
