# Supplementary material for: Assessment of risk factors in persistent subsolid pulmonary nodules based on CT dynamic follow-up
Source: BMC Med Imaging. 2026 Aug 20;26:414. doi: 10.1186/s12880-026-02694-5 (PMC13495464; doi:10.1186/s12880-026-02694-5)
Supplement: Supplementary file 1 [file 12880_2026_2694_MOESM1_ESM.docx]

**Supplementary Table S1. Characteristics of growing and non-growing GGNs and PSNs in the NLST dataset**

| Characteristics | GGN | | | PSN | | |
| --- | --- | --- | --- | --- | --- | --- |
|  | Growth group  (n=204) | Non-growth group  (n=344) | *P value* | Growth group  (n=73) | Non-growth group  (n=44) | *P value* |
| **Clinical characteristics** | | | | | | |
| Age | 62.7 (58, 74) | 61.8 (55, 74) | 0.015 | 63.4 (66, 74) | 62.5 (55, 74) | 0.365 |
| Sex |  |  | 0.754 |  |  | 0.251 |
| Female | 117 (57.4%) | 202 (58.7%) |  | 42 (57.5%) | 30 (68.2%) |  |
| male | 87 (2.6%) | 142 (41.3%) |  | 31 (42.5%) | 14 (31.8%) |  |
| Smoking history |  |  | 0.068 |  |  | 0.084 |
| Yes | 150 (73.5%) | 276 (80.2%) |  | 51 (69.9%) | 37 (84.1%) |  |
| No | 54 (26.5%) | 68 (19.8%) |  | 22 (30.1%) | 7 (15.9%) |  |
| Follow-up time | 747.1 (342.0, 995.0) | 748.3 (350.0, 1014.0) | 0.852 | 736.5 (359.0, 1026.0) | 733.1 (382.0, 904.0) | 0.657 |
| **Nodular characteristics** |  |  |  |  |  |  |
| Initial diameter (mm) | 8.5 (5.1, 24.0) | 6.8 (5.0, 21.7) | <0.001 | 10.8 (5.2, 27.6) | 9.2 (5.2, 24.3) | 0.718 |
| Initial volume (mm^3^) | 393.5 (24.5, 5188.0） | 164.5 (28.5, 3930.0) | 0.005 | 907.8 (22.5, 9432.5) | 519.3 (59.0, 3259.5) | 0.347 |
| Initial mass (mg) | 266.0 (13.6, 3861.0) | 101.0 (13.7, 3018.1) | 0.004 | 476.5 (13.0, 5469.5) | 179.5 (22.6, 1227.9) | 0.893 |
| Initial m-CTA (HU) | -374.2 (-843.2, -229.3) | -406.1 (-850.7, -271.2) | 0.002 | -464.1 (-831.4,-217.6) | -562.9 (-890.1, -354.2) | <0.001 |
| VDT (days) | 738.6 (111.4, 2299.6) |  |  | 630.2 (107.2, 1389.5) |  |  |
| MDT (days) | 1670.6 (174.7, 15439.4) |  |  | 1623.7 (262.7, 9907.6) |  |  |
| Irregular |  |  | 0.005 |  |  | 0.349 |
| No | 158 (77.5%) | 298 (86.6%) |  | 60 (82.2%) | 39 (88.6%) |  |
| Yes | 46 (22.5%) | 46 (13.4%) |  | 13 (17.8%) | 5 (11.4%) |  |
| Lesion location |  |  | 0.337 |  |  | 0.688 |
| Right upper lobe | 45 (22.1%) | 96 (27.9%) |  | 17 (23.3%) | 13 (29.5%) |  |
| Right middle lobe | 26 (12.7%) | 30 (8.7%) |  | 13 (17.8%) | 6 (13.6%) |  |
| Right lower lobe | 40 (19.6%) | 73 (21.2%) |  | 18 (24.7%) | 10 (22.7%) |  |
| Left upper lobe | 46 (22.5%) | 77 (22.4%) |  | 10 (13.7%) | 9 (20.5%) |  |
| Left lower lobe | 47 (23.0%) | 68 (19.8%) |  | 15 20.5%) | 6 (13.6%) |  |
| Morphologic features |  |  |  |  |  |  |
| Lobulated sign | 24 (11.8%) | 34 (9.9%) | 0.489 | 6 (8.3%) | 4 (9.1%) | 0.888 |
| Spiculated sign | 25 (12.3%) | 30 (8.7%) | 0.183 | 10 (13.7%) | 3 (6.8%) | 0.251 |
| Pleural indentation | 46 (22.5%) | 52 (15.1%) | 0.028 | 22 (30.1%) | 9 (20.5%) | 0.250 |
| Air bronchogram | 23 (11.3%) | 18 (5.2%) | 0.010 | 10 (13.7%) | 5 (11.4%) | 0.714 |
| Vacuole sign | 11 (5.4%) | 22 (6.4%) | 0.633 | 8 (11.0%) | 4 (9.1%) | 0.747 |

**Supplementary Table S2. Characteristics of growing and non-growing GGNs and PSNs in the Institutional dataset**

| Characteristics | GGN | | | PSN | | |
| --- | --- | --- | --- | --- | --- | --- |
|  | Growth group  (n=45) | Non-growth group  (n=73) | *P value* | Growth group  (n=37) | Non-growth group  (n=21) | *P value* |
| **Clinical characteristics** | | | | | | |
| Age | 57.0 (19, 73) | 51.1 (25, 81) | 0.001 | 58.9 (43,73) | 55.4 (33,63) | 0.151 |
| Sex |  |  | 0.154 |  |  | 0.136 |
| Female | 23 (51.1%) | 47 (64.4%) |  | 25 (67.6%) | 10 (47.6%) |  |
| male | 22 (48.9%) | 26 (35.6%) |  | 12 (32.4%) | 11 (52.4%) |  |
| Smoking history |  |  | <0.001 |  |  | 0.136 |
| Yes | 21 (46.7%) | 57 (78.1%) |  | 19 (51.4%) | 15 (71.4%) |  |
| No | 24 (53.3%) | 16 (21.9%) |  | 18 (48.6%) | 6 (28.6%) |  |
| Follow-up time | 482.7 (189.0, 1462.0) | 481.6 (185.0, 1490.0) | 0.656 | 578.3 (193.0, 1665.0) | 503.8 (193.0, 985.0) | 0.599 |
| **Nodular characteristics** |  |  |  |  |  |  |
| Initial diameter (mm) | 8.1 (5.8, 13.6) | 8.4（5.5, 11.3） | 0.842 | 10.6 (5.7, 21.1) | 9.9 (5.8, 14.7) | 0.428 |
| Initial volume (mm^3^) | 302.3 (99.7, 1036.6) | 219.2 (95.2, 716.6) | 0.135 | 714.4 (69.3, 2793.4) | 524.3 (94.4, 1475.3) | 0.324 |
| Initial mass (mg) | 101.1 (9.6, 590.4) | 76.8 (26.7, 432.0) | 0.261 | 341.4 (25.4,1396.6) | 263.3 (37.3,1150.8) | 0.477 |
| Initial m-CTA (HU) | -644.2 (-809.4, -260.2) | -661.2 (-812.3, -464.1) | 0.483 | -543.5 (-691.1, -292.0) | -546.1 (-694.4, -342.0) | 0.981 |
| VDT (days) | 915.1 (191.3, 4013.6) |  |  | 820.0 (237.8, 2290.9) |  |  |
| MDT (days) | 1346.7 (236.0, 5871.5) |  |  | 1398.1 (306.5, 9808.1) |  |  |
| Irregular |  |  | 0.765 |  |  | 0.534 |
| No | 40 (88.9%) | 66 (90.4%) |  | 18 (48.6%) | 12 (57.1%) |  |
| Yes | 5 (11.1%) | 7 (9.6%) |  | 19 (51.4%) | 9 (42.9%) |  |
| Lesion location |  |  | 0.476 |  |  | 0.231 |
| Right upper lobe | 12 (26.7%) | 18 (24.7%) |  | 11 (29.7%) | 7 (33.3%) |  |
| Right middle lobe | 8 (17.8%) | 7 (9.6%) |  | 3 (100.0%) | 0 (0.0%) |  |
| Right lower lobe | 8 (17.8%) | 21 (28.8%) |  | 3 (8.1%) | 4 (19.0%) |  |
| Left upper lobe | 9 (20.0%) | 16 (21.9%) |  | 14 (37.8%) | 6 (28.6%) |  |
| Left lower lobe | 8 (17.8%) | 11 (15.1%) |  | 6 (16.2%) | 4 (19.0%) |  |
| Morphologic features |  |  |  |  |  |  |
| Lobulated sign | 14 (31.1%) | 8 (11.0%) | 0.006 | 23 (62.2%) | 11 (52.4%) | 0.467 |
| Spiculated sign | 14 (31.1%) | 4 (5.5%) | <0.001 | 7 (18.9%) | 4 (19.0%) | 0.990 |
| Pleural indentation | 17 (37.8%) | 14 (19.2%) | 0.026 | 16 (43.2%) | 8 (38.1%) | 0.702 |
| Air bronchogram | 8 (17.8%) | 4 (5.5%) | 0.067 | 9 (24.3%) | 2 (9.5%) | 0.296 |
| Vacuole sign | 4 (8.9%) | 6 (8.2%) | 0.899 | 7 (18.9%) | 1 (4.8%) | 0.269 |
